# Gender differences in diabetes knowledge, glycemic control, and cardiovascular risk among adults with type 2 diabetes: a cross-sectional study

**DOI:** 10.3389/fendo.2026.1763252

**Published:** 2026-03-13

**Authors:** Osama Albasheer, Mohamed Salih Mahfouz, Ayyub Alssum, Mohammed Majrashi, Areej Hamdi, Wedad M. Alhazmi, Bayan Hassan Hakami, Aisha A. Awaf, Rania Hassan, Norah Rajeh, Taif Solan, Tahani Madkhali, Hatim Alessa, Afnan Madkhali, Afaf Hakami

**Affiliations:** 1 Family Medicine, Family and Community Medicine Department, Faculty of Medicine, Jazan University, Jazan, Saudi Arabia; 2 Family Medicine, Primary Healthcare, Ministry of Health, Jazan, Saudi Arabia; 3 Mawrah Primary Health Center, Southern Health Sector – Jazan, Ministry of Health, Jazan, Saudi Arabia; 4 Family Medicine, Diabetic center of King Fahad Central Hospital Jazan Ministry of Health, Jazan, Saudi Arabia; 5 Family Medicine, Jazan Health Cluster, Ministry of Health, Jazan, Saudi Arabia; 6 Adult Endocrinology and Diabetes Department, Jazan Endocrinology and Diabetes Center, Ministry of Health, Jazan, Saudi Arabia; 7 Faculty of Medicine, Jazan University, Jazan, Saudi Arabia; 8 Department of Family Medicine, Jazan University Hospital, Jazan University, Jazan, Saudi Arabia

**Keywords:** diabetic knowledge, framingham risk score, gender differences, glycemic control, primary health care, Saudi Arabia

## Abstract

**Background:**

Gender differences in diabetes knowledge, glycemic control, and cardiovascular risk remain an important public health concern. This study examined gender differences in diabetes knowledge, glycemic control, and cardiovascular risk among adults with type 2 diabetes in Saudi Arabia, with sociodemographic characteristics, lifestyle behaviors, healthcare utilization, and perceptions of recent social reforms explored as contextual factors.

**Methods:**

A cross-sectional study was conducted among 336 adults with type 2 diabetes attending primary healthcare centers. Data were collected using a structured questionnaire assessing diabetes knowledge, healthcare utilization, lifestyle behaviors, and perceptions of social reforms. Clinical data included HbA1c and Framingham Risk Score (FRS) variables. Logistic regression models were developed separately for males and females.

**Results:**

Women demonstrated higher diabetes knowledge than men (59.5% *vs*. 44.6%, p = 0.016). Among men, university education or higher was associated with substantially greater odds of appropriate knowledge compared with illiteracy (adjusted OR = 7.81, 95% CI: 1.99–30.72; p = 0.003), while among women the association remained significant but of smaller magnitude (adjusted OR = 3.93, 95% CI: 1.52–10.14; p = 0.005). Younger age was independently associated with better knowledge in both genders; participants aged 18–40 years had markedly higher odds compared with those aged >60 years (men: OR = 12.67, 95% CI: 2.67–60.05; p = 0.001; women: OR = 11.43, 95% CI: 3.79–34.51; p < 0.001). Most participants had suboptimal glycemic control, with 47.9% showing HbA1c >8% and only 19.9% achieving HbA1c <7% (p=0.889). Physical inactivity was highly prevalent, with 55.7% never engaging in exercise, and significant gender differences were observed in exercise frequency (p<0.001). Smoking was markedly higher among males (p<0.001) and contributed to higher FRS categories in men.

**Conclusion:**

Although most participants reported easy access to healthcare services, gender differences in diabetes knowledge and cardiovascular risk remained evident, while glycemic control was suboptimal across both genders. These findings underscore the need for gender-sensitive diabetes education, targeted physical activity interventions, and routine cardiovascular risk assessment as integral components of diabetes care.

## 1 Introduction

Diabetes mellitus (DM) is one of the most prevalent chronic non-communicable diseases worldwide and a leading cause of morbidity and mortality (1–3). According to the International Diabetes Federation, an estimated 537 million adults were living with diabetes globally in 2021, a figure projected to rise to 643 million by 2030 and 783 million by 2045, with nearly half of affected individuals remaining undiagnosed (2).

This trend is particularly pronounced in the Middle East and North Africa region, where rapid urbanization, lifestyle transitions, and population aging have contributed to some of the highest diabetes prevalence rates globally (4, 5).

Saudi Arabia is among the countries most affected by this epidemic (6). In recent years, major healthcare and social reforms under Vision 2030 have expanded access to healthcare services, strengthened primary care–based chronic disease management, and reduced gender-based barriers to healthcare utilization (6, 7). These changes, including improved mobility and autonomy for women, provide an important context for reassessing gender differences in diabetes knowledge, self-management behaviors, and clinical outcomes (8). Recent studies have suggested that women in Saudi Arabia are engaging more in health-promoting activities like exercise and health screenings, supported by improved healthcare infrastructure and access to facilities (9, 10). These changes have significantly reduced the gender gap in access to healthcare services, physical exercise, and diabetes management.

Globally, evidence suggests that gender differences in diabetes outcomes are complex. Women often demonstrate higher diabetes-related knowledge and engagement in preventive behaviors than men, yet may experience poorer glycemic and cardiovascular outcomes (11–14).

In Saudi Arabia, previous studies examining diabetes knowledge and glycemic control have reported inconsistent findings regarding gender differences, and most have focused on isolated outcomes rather than integrated assessments (15, 16). Furthermore, cardiovascular risk has rarely been evaluated using validated tools such as the Framingham Risk Score within a gender-stratified framework.

This study aimed to assess gender differences in diabetes knowledge, glycemic control, and cardiovascular risk among adults with type 2 diabetes in Saudi Arabia, with sociodemographic characteristics, healthcare utilization, lifestyle behaviors, and perceptions of recent social reforms examined as contextual factors. We hypothesized that women would demonstrate higher diabetes knowledge than men, that improved healthcare access would not necessarily translate into optimal glycemic control, and that cardiovascular risk profiles would differ by gender.

## 2 Methodology

### 2.1 Study design and setting

A cross-sectional study was conducted between October 2024 and June 2025 among adults with type 2 diabetes mellitus attending selected primary healthcare centers (PHCs) in the Jazan region, Saudi Arabia. The region includes urban and semi-urban areas, and PHCs serve as the main point of care for chronic disease management, including diabetes. Data collection was carried out between.

The study was conducted in accordance with the Helsinki Declaration and Saudi Bioethics standards’ guidelines. Approval was obtained from the Jazan Health Cluster Ethics Committee, Saudi Arabia, reference number 2467, dated October 14, 2024. Informed consent was obtained from all subjects involved in the study. Participation was voluntary, and strict measures were implemented to ensure anonymity and confidentiality throughout the study. No identifiable personal information was collected or stored.

### 2.2 Study population, sampling, and sample size

Eligible participants were community-dwelling (non-institutionalized) adults aged ≥18 years with a confirmed diagnosis of type 2 diabetes mellitus who attended the selected primary healthcare centers during the study period. Individuals with type 1 diabetes, gestational diabetes, severe cognitive impairment, acute illness, or inability to provide informed consent were excluded. Both males and females were included. No restrictions were placed on physical activity level or presence of diabetes-related comorbidities to ensure representativeness of routine primary care populations.

The minimum required sample size was calculated using Epi Info™ version 7.2 (17), assuming a 95% confidence level, 5% margin of error, an expected prevalence of adequate diabetes knowledge of 50%, and a non-response rate of 20%. This yielded a target sample size of 327 participants. During implementation of the sampling plan and for practical reasons, a total of 336 participants were ultimately recruited. This final sample size was sufficient to support gender-stratified analyses and multivariable modeling.

There were 179 primary healthcare centers in the region, distributed across coastal, plains, and mountainous sub-regions. Nine centers were selected using simple random sampling. Probability proportional to size sampling was applied to allocate participant numbers across centers. Participants were recruited consecutively during routine clinic visits.

### 2.3 Data collection instruments

Data were collected using a structured interviewer-administered questionnaire and clinical record review. Instruments assessed the primary outcomes of diabetes knowledge, glycemic control, and cardiovascular risk, alongside contextual variables including sociodemographic characteristics, healthcare utilization, lifestyle behaviors, and perceptions of recent social reforms.

#### 2.3.1 Sociodemographic and clinical characteristics

Information on age, sex, marital status, education, employment, residence, and diabetes duration was collected. Glycated hemoglobin (HbA1c) values and lipid profiles were extracted from medical records. Glycemic control was categorized using standard clinical cutoffs.

Hormonal and reproductive variables were collected for female participants for descriptive purposes but were not included in the final analysis and are acknowledged as a study limitation.

#### 2.3.2 Diabetes knowledge questionnaire

The diabetes knowledge questionnaire was adapted from previously published diabetes knowledge instruments and diabetes KAP studies widely used in diabetes research, including instruments such as the Diabetes Knowledge Questionnaire (DKQ) and other KAP-based tools that assess knowledge, attitudes, and practices in people with diabetes (18, 19). The items covered standard diabetes education domains, including disease mechanisms, symptoms and complications, glycemic control, diet, physical activity, medication use, and foot care. The questionnaire was not a proprietary instrument and did not require formal permission for use.

The questionnaire was translated into Arabic using a standardized forward–backward translation procedure and was pilot-tested among 30 participants to ensure clarity and comprehensibility. Internal consistency reliability was evaluated in the present study, yielding a Cronbach’s alpha coefficient of 0.864, indicating good reliability.

Each knowledge item was scored as 1 for a correct response and 0 for an incorrect or ‘Don’t know’ response; item scores were summed and converted to a percentage score to generate the composite diabetes knowledge variable. A score ≥60% was classified as appropriate knowledge, while a score <60% indicated inappropriate knowledge, consistent with thresholds commonly used in diabetes knowledge and KAP research (20, 21).

#### 2.3.3 Healthcare utilization, lifestyle behaviors, and perceptions of social reforms

Healthcare utilization variables included frequency of clinic visits and participation in structured diabetes education programs. Lifestyle behaviors assessed included smoking status, physical activity, and dietary modification. Physical activity was evaluated based on self-reported frequency and type of exercise. Dietary modification was assessed by self-reported adherence to dietary recommendations. Perceptions regarding recent social and healthcare reforms were assessed using structured questionnaire items.

#### 2.3.4 Cardiovascular risk assessment

Cardiovascular risk was estimated using the general cardiovascular disease Framingham Risk Score as described by D’Agostino et al. (22), a validated tool that estimates the 10-year risk of developing cardiovascular disease (22). The score was calculated using age, sex, total cholesterol, high-density lipoprotein (HDL) cholesterol, systolic blood pressure, antihypertensive treatment status, smoking status, and diabetes status. Individual risk scores were derived using the standard Framingham point-based algorithm. Participants were subsequently classified into low (<10%), intermediate (10–20%), or high (>20%) 10-year cardiovascular risk categories according to established Framingham criteria. The FRS was applied uniformly across the study population to allow gender-stratified comparisons of cardiovascular risk. Although the FRS was originally developed in Western populations, it was used in this study as a validated comparative risk assessment tool, and its limitations when applied to Middle Eastern populations are acknowledged.

### 2.4 Statistical analysis

Data analysis was conducted using IBM SPSS Statistics for Windows, version 26 (IBM Corp., Armonk, N.Y., USA). Descriptive statistics, including frequencies and percentages, were used to summarize participants’ demographic characteristics, healthcare utilization, and lifestyle factors. The Chi-square test or Fisher’s exact test, as appropriate, was applied to examine associations between categorical variables such as knowledge levels and access to healthcare services. Normality testing was not required, as the primary analyses involved categorical variables and logistic regression models.

Binary logistic regression analyses were performed to identify factors independently associated with appropriate diabetes knowledge. Separate gender-stratified models were constructed for male and female participants to allow comparison of predictors across genders. For regression analyses, employment status was dichotomized as employed versus unemployed to ensure model stability. The multivariable models included age group, educational level, employment status, and diabetes duration as covariates selected *a priori* based on their clinical and epidemiological relevance. Adjusted odds ratios (ORs) with corresponding 95% confidence intervals (CIs) were reported. Statistical significance was defined as a two-sided p-value <0.05.

## 3 Results

### 3.1 Participants’ characteristics and their diabetes knowledge level

Of the 336 participants, males and females were equally represented (50% each) (**Table 1**). Knowledge levels differed significantly by gender, with females showing a higher proportion of appropriate knowledge compared with males (p = 0.016).

**Table 1:** Participants’ background information and their level of knowledge regarding diabetes and its control among the Jazan population, categorized by gender (n=336).

Characteristics		All participants	Knowledge level		p. value*
			Inappropriate	Appropriate	
		N%			
Gender	Female	168(50.0)	58(34.5)	110(65.5)	0.016
	Male	168(50.0)	38(22.6)	130(77.4)	
Age(years)	18–less than 30	19(5.7)	4(21.1)	15(78.9)	<0.001
	30–less than 40	68(20.2)	5(7.4)	63(92.6)	
	40- less than 50	75(22.3)	14(18.7)	61(81.3)	
	50- less than 60	104(31.0)	37(35.6)	67(64.4)	
	60 years and more	70(20.8)	36(51.4)	34(48.6)	
Marital Status	Divorced	38(11.3)	10(26.3)	28(73.7)	0.589
	Married	223(66.4)	68(30.5)	155(69.5)	
	Single	44(13.1)	9(20.5)	35(79.5)	
	Widowed	31(9.2)	9(29.0)	22(71.0)	
Residence	Village	141(42.0)	36(25.5)	105(74.5)	0.294
	Town	195(58.0)	60(30.8)	135(69.2)	
Education Level	Illiterate	103(30.7)	44(42.7)	59(57.3)	<0.001
	Basic Education	144(42.9)	42(29.2)	102(70.8)	
	University	81(24.1)	9(11.1)	72(88.9)	
	Postgraduate (Higher)	8(2.4)	1(12.5)	7(87.5)	
Employment Status	Own business	10(3.0)	2(20.0)	8(80.0)	<0.001#
	House Wife	89(26.5)	22(24.7)	67(75.3)	
	Student	3(.9)	0(.0)	3(100.0)	
	Laborer	10(3.0)	4(40.0)	6(60.0)	
	Unemployed	111(33.0)	49(44.1)	62(55.9)	
	Employed	113(33.6)	19(16.8)	94(83.2)	
Duration of Diabetes	Less than 1 year	24(7.1)	9(9.4)	15(6.3)	0.007
	1–5 years	62(18.5)	22(22.9)	40(16.7)	
	6–10 years	77(22.9)	26(27.1)	51(21.3)	
	10–15 years	81(24.1)	10(10.4)	71(29.6)	
	More than 15 years	92(27.4)	29(30.2)	63(26.3)	
Most Recent HbA1c Level	Above 8%	161(47.9)	44(27.3)	117(72.7)	0.889
	7%–8%	108(32.1)	32(29.6)	76(70.4)	
	Below 7%	67(19.9)	20(29.9)	47(70.1)	

*p. value is based on Chi Squared test, #= p value based on Fisher Exact test as the assumptions of Chi Squared test were not met; HbA1c, Hemoglobin A1c.

The largest age group consisted of participants aged 50–59 years (31%), followed by those aged 40–49 years (22.3%) and those aged 60 years or older (20.8%). Younger age groups demonstrated significantly higher proportions of appropriate knowledge (p < 0.001).

Most participants were married (66.4%), and the majority resided in towns (58%). In terms of education, basic education was the most common level attained (42.9%), followed by illiteracy (30.7%). A smaller proportion held university degrees (24.1%) or postgraduate qualifications (2.4%). University or higher educational attainment was strongly associated with appropriate knowledge (p < 0.001).

Regarding employment status, 33.6% of participants were employed, while 33% were unemployed. Employed individuals had a significantly higher proportion of appropriate knowledge, whereas unemployed participants showed the highest proportion of inappropriate knowledge (p < 0.001).

Participants with more than 15 years of diabetes constituted the largest duration category (27.4%), followed by those with 10–15 years (24.1%) and 6–10 years (22.9%). Knowledge levels varied significantly across duration categories (p = 0.007).

As for glycemic control, 47.9% of participants had an HbA1c level above 8%, while 19.9% had HbA1c levels below 7%. No statistically significant association was observed between diabetes knowledge level and glycemic control as measured by HbA1c categories (p = 0.889).

Detailed item-level responses to the diabetes knowledge statements are presented in **Supplementary Table 1**. The table shows the distribution of “Yes,” “No,” and “Don’t know” responses for each knowledge item stratified by gender and forms the basis for the composite diabetes knowledge categories summarized in **Table 1**. Items were grouped into conceptual domains based on diabetes education frameworks to enhance interpretability; domain classification was applied for descriptive purposes only. Across most items, response patterns were similar between males and females, with no statistically significant gender differences observed at the individual item level (all p > 0.05).

### 3.2 Participants’ healthcare utilization, lifestyle behaviors, and perceptions related to recent social reforms

Regarding healthcare utilization, 43.2% of participants reported visiting a healthcare provider every 2–3 months, followed by 26.8% who attended monthly, 19.6% every six months, and 1.5% annually, while 3.6% reported irregular attendance (**Table 2**). There was no significant gender difference in healthcare utilization patterns (p > 0.05). Participation in diabetes education programs was low, with only 16.7% reporting attendance, and this did not differ significantly between males and females (p > 0.05). Additionally, a large majority (84.2%) stated that accessing healthcare services was easy.

**Table 2:** Participants perception regarding lifestyle, health-promoting activities and social reforms stratified by gender (n=336).

Factors		All participants	Male	Female	p. value*
		N%			
Healthcare utilization	Once a month	90(26.8)	42(25.0)	48(28.6)	0.540#
	Every 2–3 months	163(48.5)	78(46.4)	85(50.6)	
	Every 6 months	66(19.6)	39(23.2)	27(16.1)	
	Once a year	5(1.5)	3(1.8)	2(1.2)	
	Not regularly	12(3.6)	6(3.6)	6(3.6)	
Participation in diabetes education	Yes	77(22.9)	37(22.0)	40(23.8)	0.698#
	No	258(76.8)	131(78.0)	127(75.6)	
	Don’t Know	1(.3)	0(.0)	1(.6)	
Easy access to healthcare services	Yes	291(86.6)	146(86.9)	145(86.3)	0.873
	No	45(13.4)	22(13.1)	23(13.7)	
Engagement in physical exercise	1–2 times/week	91(27.1)	52(31.0)	39(23.2)	<0.001
	3–4 times/week	44(13.1)	18(10.7)	26(15.5)	
	≥ 5 times/week	29(8.6)	24(14.3)	5(3.0)	
	Never	172(51.2)	74(44.0)	98(58.3)	
Dietary modification	Yes	253(75.3)	131(78.0)	122(72.6)	0.255
	No	83(24.7)	37(22.0)	46(27.4)	
Perceived impact of social reforms	Yes	200(59.5)	105(62.5)	95(56.5)	0.198
	No	56(16.7)	30(17.9)	26(15.5)	
	Don’t Know	80(23.8)	33(19.6)	47(28.0)	
Equal access to healthcare services	Yes	262(78.0)	131(78.0)	131(78.0)	0.500
	No	31(9.2)	18(10.7)	13(7.7)	
	Don’t Know	43(12.8)	19(11.3)	24(14.3)	

*p value is based on Chi Squared test, #= p value based on Fisher Exact test as the assumptions of Chi Squared test were not met.

Lifestyle behaviors varied across participants. Approximately half of participants (51.2%) reported never engaging in physical exercise, while 13.1% reported exercising 2–3 times per week, with a significant gender difference observed in exercise frequency (p < 0.001). Among participants who engaged in physical activity, walking was the most commonly reported exercise type (84.2%). Most participants rated their physical activity level as low (16.7%) or very low (44.3%), with no significant gender difference in activity level categories (p = 0.238).

Dietary modification was reported by 74.1% of participants, with no significant difference between males and females (p > 0.05).

With respect to the perceived impact of social reforms, 56.8% of participants felt that recent changes improved their ability to manage diabetes, while 52.4% believed that these reforms made it easier to engage in physical activities. Furthermore, 71.1% stated that they now have equal access to healthcare services compared with the opposite gender. None of these perceptions differed significantly between males and females (all p > 0.05).

### 3.3 Gender difference and the distribution of the cardiovascular risk factors

Smoking status showed a clear gender difference, with a significantly higher proportion of males reporting current (23.2%) or former smoking (26.8%) compared with females, among whom 94.6% were non-smokers (p < 0.001) (**Table 3**).

**Table 3:** Cardiovascular risk factors (Framingham Risk components) according to gender among the study population (n=336).

Variables		Gender N(%)		p. value*
		Male	Female	
Smoking status	Current smoker	39(23.2)	7(4.2)	<.001
	Former smoker	45(26.8)	2(1.2)	
	Never smoked	84(50.0)	159(94.6)	
Current blood pressure (in mmHg) level	≥ 140/90	43(25.6)	60(35.7)	.109
	120/80 -139/89	62(36.9)	58(34.5)	
	< 120/80	63(37.5)	50(29.8)	
Current antihypertensive	Yes	95(56.5)	69(41.1)	.659
	No	73(43.5)	99(58.9)	
Most recent total cholesterol level (in mg/dl)	200–239	49(29.2)	70(41.7)	.055
	≥ 240	25(14.9)	22(13.1)	
	< 200	94(56.0)	76(45.2)	
Most recent HDL level (if (in mg/dl)	40–59	78(46.4)	75(44.6)	.917
	≥60	38(22.6)	41(24.4)	
	<40	52(31.0)	52(31.0)	
Current medication for cholesterol management	Yes	109(64.9)	108(64.3)	.909
	No	59(35.1)	60(35.7)	
Family History of Cardiovascular Disease or stroke	Yes	88(52.4)	66(39.3)	.016
	No	80(47.6)	102(60.7)	

*p. value is based on Chi Squared test.

Blood pressure levels were similarly distributed across genders. One-third of participants reported elevated systolic blood pressure, with no significant gender difference (p > 0.05). Use of antihypertensive medication was also comparable between males and females (p > 0.05).

Total cholesterol levels showed minor variation, with more females reporting levels between 200–239 mg/dL, while a slightly higher proportion of males had levels below 200 mg/dL; these differences were not statistically significant (p > 0.05).

HDL cholesterol categories were similarly distributed across genders, with no significant difference in the prevalence of low HDL levels (p > 0.05). Statin use was also nearly identical between males and females (p > 0.05).

Family history of cardiovascular disease showed a gender difference, with males reporting a higher frequency of positive family history compared with females (p < 0.05).

### 3.4 Logistic regression models examining factors associated with appropriate diabetes awareness separately for males and females

Among males, education level was a strong predictor of appropriate knowledge (**Table 4**). Participants with university education had significantly higher odds of appropriate knowledge compared with illiterate participants (p < 0.001). Age was also a significant factor; males younger than 50 years demonstrated higher odds of appropriate knowledge compared with those aged 60 years or older (p < 0.05). Employment status and duration of diabetes were not significantly associated with knowledge (p > 0.05). Participation in diabetes education programs showed a borderline association in the male model but did not reach statistical significance.

**Table 4:** Logistic regression analysis of the factors associated with awareness of diabetes control among the study participants.

Category	Male model				Female model			
	p. value	OR	95% C.I.		p. value	OR	95% C.I.	
Education								
Illiterate (Ref.)		1				1		
Basic Education	0.742	1.16	0.49	2.74	0.029	2.24	1.09	4.62
University education or higher	0.003	7.81	1.99	30.72	0.005	3.93	1.52	10.14
Age (years)								
18–40 years	0.001	12.67	2.67	60.05	<0.001	11.43	3.79	34.51
41–60 years	0.046	2.27	1.01	5.07	0.002	3.87	1.62	9.28
More than 60 years (Ref.)		1				1		
Work status								
Unemployed (Ref.)		1				1		
Employed	0.248	1.54	0.74	3.19	0.022	3.28	1.19	9.07
Duration of diabetes								
5 years or less (Ref.)		1				1		
More than 5 years	0.405	1.37	0.66	2.84	0.060	2.08	0.97	4.48
Participation in any diabetes education programs?								
Yes	0.060	2.08	0.97	4.48	0.405	1.37	0.66	2.84
No (Ref.)		1				1		

REF, references; OR, odds ratio; CI, confidence interval.

Among females, both basic and university education were significantly associated with higher odds of appropriate knowledge compared with illiteracy (p < 0.001). Younger age groups also had significantly greater odds of appropriate awareness than those aged 60 years or older (p < 0.05). Employment status was positively associated with appropriate knowledge (p < 0.05). In contrast, disease duration and participation in diabetes education programs were not significantly associated with knowledge in the female model (p > 0.05).

## 4 Discussion

### 4.1 Main findings

The present study focused on identifying predictors of diabetes knowledge, while glycemic control and cardiovascular risk were described to contextualize the clinical status of the study population. Women demonstrated higher diabetes knowledge than men; however, both genders exhibited suboptimal glycemic control, elevated cardiovascular risk, and very low levels of physical activity. These findings are consistent with international literature reporting a persistent gap between diabetes knowledge and clinical outcomes among individuals with type 2 diabetes.

### 4.2 Diabetes knowledge and sociodemographic differences

Women in this study showed higher levels of diabetes knowledge than men. This observation is consistent with several international reports indicating that women tend to engage more frequently in health information–seeking and preventive behaviors (11, 13). Similar trends have been reported where women often show higher health literacy or health knowledge and are more engaged with health information (14, 23).

Despite moderate overall knowledge scores, item-level analysis revealed persistent misconceptions across both genders, particularly regarding insulin physiology and the differentiation between symptoms of hypoglycemia and hyperglycemia. This indicates that composite knowledge measures may obscure clinically relevant gaps in practical diabetes understanding. Comparable patterns have been reported in regional and international studies, where patients demonstrated acceptable general awareness yet retained incorrect beliefs related to core pathophysiological concepts and symptom recognition. These findings emphasize the importance of complementing global knowledge assessments with targeted educational strategies that address specific conceptual errors critical to daily diabetes self-management and the prevention of acute complications.

However, several global studies, such as NHANES (USA) and the DAWN2 international study (involving 17 countries), have demonstrated universally low diabetes knowledge regardless of gender (24, 25). Studies across Asia, including in India (26), China (27), and Malaysia (28), have consistently shown substantial diabetes knowledge gaps, particularly among older adults and individuals with low educational attainment. In line with these findings, younger age and higher education were associated with better diabetes knowledge in the present study, indicating a global pattern where education and age significantly shape diabetes literacy.

Employment status was associated with higher diabetes knowledge among women. Evidence regarding this relationship has been inconsistent. A workplace-focused systematic review reported that health literacy among employees with diabetes was often lower than optimal, leading to missed opportunities for effective diabetes management, although the review did not assess gender differences (29). In addition, a Korean gender-employment analysis found that women with type 2 diabetes had significantly lower employment rates than women without diabetes and lower employment probabilities compared with men, indicating that diabetes may reduce women’s workforce participation rather than reflect enhanced literacy (30). The positive association between employment and diabetes knowledge among women in this study may reflect the unique socio-cultural context in Saudi Arabia, where employed women tend to have higher education, greater autonomy, and more exposure to health information through workplace settings.

### 4.3 Healthcare utilization and diabetes education

Most participants reported regular healthcare follow-up and perceived healthcare access as easy. This observation aligns with recent expansions in primary healthcare services under Vision 2030 (6, 7). A notable finding is that most participants believed they now have equal access to healthcare services regardless of gender—reflecting the impact of major social reforms implemented since 2018, including women’s ability to drive and greater mobility. It is important to note that recent social and legal reforms were not directly measured or analytically tested in this study and are therefore discussed as contextual factors rather than determinants of diabetes outcomes. Globally, regular follow-up remains essential for optimal diabetes control, with recent meta-analyses demonstrating that participation in diabetes self-management education and timely clinic visits are associated with improved glycemic and cardiovascular outcomes (31, 32). Despite this, attendance at structured diabetes education programs in our sample was very low. This pattern aligns with international evidence showing that uptake of formal diabetes self-management education remains sub-optimal in many countries (33, 34), representing a persistent challenge in diabetes care delivery. Regions with high participation in structured diabetes education—particularly where national programs are standardized and widely implemented—have consistently demonstrated better diabetes knowledge, improved glycemic control, enhanced self-management behaviors, and lower rates of cardiovascular complications (35).

### 4.4 Lifestyle behaviors and physical activity

Physical inactivity was highly prevalent, with most participants reporting low or no engagement in exercise. This mirrors national epidemiological surveys indicating that physical inactivity remains widespread among Saudi adults, especially those with chronic diseases (36). The extremely low physical activity levels observed in this study also reflect global patterns. The WHO global pooled analysis indicates that around one-third of adults worldwide have insufficient physical activity (37), with many studies showing even higher inactivity levels among people with type 2 diabetes compared with the general population (38, 39). Even though many participants acknowledged that social reforms made physical activity more accessible, this perception did not translate into actual behavioral change. Walking was the most common activity in this cohort, a pattern echoed in some Asian studies where walking is often the primary form of exercise among people with type 2 diabetes (40, 41).

Dietary modification was frequently reported by participants, but global research indicates that self-reported dietary adherence is often overestimated. Studies in Europe, the US, and Asia have shown that knowledge does not necessarily translate into better diet quality or glycemic control (42–44). Our findings reinforce this global “knowledge–behavior gap”.

### 4.5 Glycemic control and cardiovascular risk

In this study, higher diabetes knowledge was not significantly associated with better glycemic control. This finding should be interpreted cautiously, as the cross-sectional design precludes causal inference and does not account for factors such as treatment adherence, disease severity, or therapeutic inertia. This pattern is consistent with global evidence: long-term follow-up of the UK Prospective Diabetes Study (UKPDS) demonstrated that even with structured and intensive management, maintaining optimal glycaemia over time remains challenging due to progressive β-cell decline and treatment fatigue (45). Large observational studies from developing countries (46) and Latin America (47) indicate that many patients with type 2 diabetes remain uncontrolled despite routine care, highlighting the persistent gap between knowledge, clinical follow-up, and metabolic outcomes.

In terms of cardiovascular risk, our cohort showed a substantial burden of risk factors, especially among men, who were more likely to smoke. This pattern aligns with Saudi and regional literature, where male patients with diabetes consistently exhibit higher smoking rates and more pronounced clustering of metabolic risk factors (48). A large meta-analysis of 74 epidemiological studies, including over 3 million individuals with type 2 diabetes from 33 countries, found that current smoking was almost five times more prevalent among men than women with diabetes (49), reinforcing the gendered distribution of cardiovascular risk behaviors.

Regional studies from the Middle East, South Asia, and Eastern Europe suggest that when tools such as the Framingham Risk Score are applied, a substantial proportion of adults—particularly those with type 2 diabetes—fall into intermediate or high cardiovascular-risk categories, underscoring the heavy atherosclerotic burden in these populations (50–52). Our results align with these regional trends and underscore the importance of routine cardiovascular risk assessment—using validated tools such as the Framingham Risk Score—as an essential component of diabetes management. Accordingly, the Framingham Risk Score findings in this study should be interpreted as reflecting relative cardiovascular risk patterns and gender differences rather than precise absolute risk estimates, given the known limitations of this tool in Middle Eastern populations.

### 4.6 Gender differences in diabetes outcomes

Globally, gender differences in diabetes outcomes are complex. Several large studies have shown that women specially younger women, with diabetes often experience worse cardiovascular outcomes than men, despite generally having better health knowledge and adopting healthier behaviors (53). Previous Saudi studies have shown mixed findings in relation to gender difference and glycemic control (15, 16). In this study, knowledge was higher among women but did not translate into better glycemic control or risk profile. This paradox has been attributed to biological and hormonal factors, delayed diagnosis, and more aggressive progression of cardiovascular disease in women with diabetes.

### 4.7 Strengths and limitations

This study has several notable strengths. First, it provides a gender-stratified analysis of diabetes knowledge, lifestyle behaviors, glycemic control, and cardiovascular risk—an approach rarely explored in Saudi Arabia despite ongoing social and healthcare reforms. Second, the study incorporates both behavioral indicators (knowledge, lifestyle practices, healthcare utilization) and clinical risk assessment using the Framingham Cardiovascular Risk Score, enabling a more holistic understanding of diabetes management patterns. Third, the sample size was sufficient to allow meaningful subgroup comparisons and logistic regression analysis, strengthening the validity of identified predictors. Fourth, the study captured patient perceptions of recent social reforms, offering a novel perspective not commonly examined in diabetes literature, either regionally or globally. Finally, gender-specific models enabled clearer differentiation between educational, biological, and socioeconomic contributors to diabetes awareness.

Several limitations must be considered when interpreting the findings. The cross-sectional design limits causal inference regarding the relationship between knowledge, behaviors, and glycemic or cardiovascular outcomes. In addition, the influence of recent social and legal reforms was not operationalized as a measurable exposure, limiting causal inference regarding their role in observed gender differences. Self-reported data on variables such as physical activity, dietary adherence, and healthcare utilization may be prone to recall or social desirability bias. The Framingham Risk Score, although widely used, was developed in Western populations and may not perfectly reflect cardiovascular risk in Middle Eastern populations with high background rates of diabetes, obesity, and early-onset metabolic syndrome. Additionally, the study was conducted in a single region of Saudi Arabia, which may limit generalizability to more diverse geographic or socioeconomic contexts. Participation in structured diabetes education programs was low, limiting the ability to fully assess their potential impact. Several potential confounders were not fully accounted for in the adjusted analyses. Variables such as body mass index, specific antidiabetic medication regimens, and treatment intensity may influence diabetes knowledge, glycemic control, and cardiovascular risk but were not included due to data limitations. In addition, diabetes duration was categorized and may not fully capture disease severity or treatment complexity and the interaction effects between gender and sociodemographic variables (e.g., education or employment) were not formally tested. These factors should be considered when interpreting the findings and warrant further investigation in future studies with more comprehensive clinical data. The study was not powered to detect small gender differences at the individual knowledge-item level, and the absence of statistical significance in these comparisons may reflect limited power and potential Type II error rather than true equivalence. Finally, although the study examined gender differences, it did not incorporate biological markers such as hormonal profiles, menopausal status, or psychosocial metrics (e.g., depression, stress), which could further clarify gender-specific patterns.

### 4.8 Future directions

Future research should consider longitudinal or cohort designs to evaluate how knowledge, lifestyle behaviors, and healthcare utilization evolve over time and how they influence long-term clinical outcomes. Incorporating biological and hormonal markers may help explain persistent gender differences in glycemic and cardiovascular risk that are not fully accounted for by healthcare access or behavioral variables. Qualitative or mixed-methods studies may also provide deeper insight into barriers to physical activity, dietary adherence, and engagement with diabetes education programs. Additionally, randomized or interventional studies should evaluate the effectiveness of structured diabetes self-management education programs tailored to older adults, individuals with low literacy, and unemployed populations—groups identified as having lower awareness. Given the rapid progression of social and legal reforms in Saudi Arabia, future studies should also explore how changes in women’s mobility, workforce participation, and digital health use impact diabetes outcomes across different regions of the Kingdom. Finally, examining alternative or locally calibrated cardiovascular risk engines may provide a more accurate assessment for Middle Eastern populations.

## 5 Conclusion

This study highlights gender differences in diabetes knowledge among adults with type 2 diabetes in Saudi Arabia, with women demonstrating higher levels of awareness than men. However, despite these differences in knowledge, both genders exhibited suboptimal glycemic control, low levels of physical activity, and a high burden of cardiovascular risk factors. Younger age, higher educational attainment, and employment status were associated with better diabetes knowledge, underscoring the role of socioeconomic factors in shaping health awareness.

Although many participants perceived improvements in healthcare access and lifestyle opportunities within the current social and healthcare context, these perceptions were not accompanied by optimal behavioral or metabolic outcomes. Given the cross-sectional design, no causal inferences can be made regarding the influence of recent social or legal reforms on diabetes management or cardiovascular risk.

Overall, the findings emphasize the need for contextually appropriate, gender-sensitive diabetes education and lifestyle support strategies that address persistent gaps between knowledge, behavior, and clinical outcomes. Future longitudinal and interventional studies are warranted to evaluate how healthcare utilization patterns, lifestyle behaviors, biological factors, and evolving social environments jointly influence diabetes outcomes among men and women in Saudi Arabia.

## Data Availability

The original contributions presented in the study are included in the article/**Supplementary Material**. Further inquiries can be directed to the corresponding author.
