# Supplementary material for: Gender differences in diabetes knowledge, glycemic control, and cardiovascular risk among adults with type 2 diabetes: a cross-sectional study
Source: Front Endocrinol (Lausanne). 2026 Mar 13;17:1763252. doi: 10.3389/fendo.2026.1763252 (PMC13021489; doi:10.3389/fendo.2026.1763252)
Supplement: Supplementary file 1 [file Table1.docx]

**Supplementary Table S1. Item-Level Responses to Diabetes Knowledge Statements by Gender (n = 336)**

| **Statements** | **Response** | | **All participants** | | **Male** | | **Female** | | ***p*. value*** |
| --- | --- | --- | --- | --- | --- | --- | --- | --- | --- |
|  |  |  | **N** | **%** | **N** | **%** | **N** | **%** |  |
| **Domain 1: Disease Mechanism and Physiology** | | | | | | | | | |
| Diabetes happens when the body has trouble controlling sugar in the blood | | **Yes** | 232 | (69.0) | 121 | (72.0) | 111 | (66.1) | .465 |
|  |  | No | 40 | (11.9) | 19 | (11.3) | 21 | (12.5) |  |
|  |  | Don't Know | 64 | (19.0) | 28 | (16.7) | 36 | (21.4) |  |
| Kidneys produce insulin. | | Yes | 37 | (11.0) | 16 | (9.5) | 21 | (12.5) | .239 |
|  |  | **No** | 209 | (62.2) | 112 | (66.7) | 97 | (57.7) |  |
|  |  | Don't Know | 90 | (26.8) | 40 | (23.8) | 50 | (29.8) |  |
| **Domain 2: Symptoms and Acute adverse events** | | | | | | | | | |
| Frequent urination and thirst are signs of low blood sugar. | | Yes | 45 | (13.4) | 19 | (11.3) | 26 | (15.5) | .467 |
|  |  | **No** | 257 | (76.5) | 133 | (79.2) | 124 | (73.8) |  |
|  |  | Don't Know | 34 | (10.1) | 16 | (9.5) | 18 | (10.7) |  |
| Shaking and sweating are signs of high blood sugar. | | Yes | 30 | (9.0) | 16 | (9.5) | 14 | (8.5) | .681 |
|  |  | **No** | 275 | (82.6) | 140 | (83.3) | 135 | (81.8) |  |
|  |  | Don't Know | 28 | (8.4) | 12 | (7.1) | 16 | (9.7) |  |
| An insulin reaction is caused by too much food. | | Yes | 72 | (21.4) | 40 | (23.8) | 32 | (19.0) | .459 |
|  |  | **No** | 158 | (47.0) | 74 | (44.0) | 84 | (50.0) |  |
|  |  | Don't Know | 106 | (31.5) | 54 | (32.1) | 52 | (31.0) |  |
| **Domain 3: Monitoring and Treatment Principles** | | | | | | | | | |
| The best way to check my diabetes is by testing my urine. | | Yes | 33 | (9.8) | 14 | (8.3) | 19 | (11.3) | .135 |
|  |  | **No** | 261 | (77.7) | 138 | (82.1) | 123 | (73.2) |  |
|  |  | Don't Know | 42 | (12.5) | 16 | (9.5) | 26 | (15.5) |  |
| A fasting blood sugar level of 210 is too high. | | **Yes** | 294 | (87.5) | 150 | (89.3) | 144 | (85.7) | .528 |
|  |  | No | 26 | (7.7) | 12 | (7.1) | 14 | (8.3) |  |
|  |  | Don't Know | 16 | (4.8) | 6 | (3.6) | 10 | (6.0) |  |
| A diabetic diet consists mostly of special foods. | | Yes | 290 | (86.3) | 145 | (86.3) | 145 | (86.3) | .111 |
|  |  | **No** | 19 | (5.7) | 13 | (7.7) | 6 | (3.6) |  |
|  |  | Don't Know | 27 | (8.0) | 10 | (6.0) | 17 | (10.1) |  |
| Medication is more important than diet and exercise to control my diabetes. | | Yes | 96 | (28.6) | 51 | (30.4) | 45 | (26.8) | .161 |
|  |  | **No** | 215 | (64.0) | 109 | (64.9) | 106 | (63.1) |  |
|  |  | Don't Know | 25 | (7.4) | 8 | (4.8) | 17 | (10.1) |  |
| **Domain 4: Lifestyle and Daily Self-Management** | | | | | | | | | |
| The way I prepare my food is as important as the foods I eat. | | **Yes** | 257 | (76.5) | 125 | (74.4) | 132 | (78.6) | .451 |
|  |  | No | 26 | (7.7) | 16 | (9.5) | 10 | (6.0) |  |
|  |  | Don't Know | 53 | (15.8) | 27 | (16.1) | 26 | (15.5) |  |
| Tight elastic hose or socks are not bad for diabetics. | | Yes | 105 | (31.3) | 49 | (29.2) | 56 | (33.3) | .477 |
|  |  | **No** | 147 | (43.8) | 79 | (47.0) | 68 | (40.5) |  |
|  |  | Don't Know | 84 | (25.0) | 40 | (23.8) | 44 | (26.2) |  |
| **Domain 5: Chronic Complications and Prevention** | | | | | | | | | |
| Diabetes can cause loss of feeling in my hands, fingers, and feet. | | **Yes** | 299 | (89.0) | 148 | (88.1) | 151 | (89.9) | .836 |
|  |  | No | 14 | (4.2) | 8 | (4.8) | 6 | (3.6) |  |
|  |  | Don't Know | 23 | (6.8) | 12 | (7.1) | 11 | (6.5) |  |
| Diabetes can damage my kidneys. | | **Yes** | 306 | (91.1) | 152 | (90.5) | 154 | (91.7) | .913# |
|  |  | No | 9 | (2.7) | 5 | (3.0) | 4 | (2.4) |  |
|  |  | Don't Know | 21 | (6.3) | 11 | (6.5) | 10 | (6.0) |  |
| Diabetes often causes poor circulation. | | **Yes** | 270 | (80.4) | 136 | (81.0) | 134 | (79.8) | .879 |
|  |  | No | 17 | (5.1) | 9 | (5.4) | 8 | (4.8) |  |
|  |  | Don't Know | 49 | (14.6) | 23 | (13.7) | 26 | (15.5) |  |
| In untreated diabetes, the amount of sugar in the blood usually increases. | | **Yes** | 306 | (91.1) | 151 | (89.9) | 155 | (92.3) | .651# |
|  |  | No | 10 | (3.0) | 5 | (3.0) | 5 | (3.0) |  |
|  |  | Don't Know | 20 | (6.0) | 12 | (7.1) | 8 | (4.8) |  |
| **Domain 6: Foot Care and Wound Management** | | | | | | | | | |
| A person with diabetes should cleanse a cut with iodine and alcohol. | | Yes | 246 | (73.2) | 122 | (72.6) | 124 | (73.8) | .875 |
|  |  | **No** | 39 | (11.6) | 21 | (12.5) | 18 | (10.7) |  |
|  |  | Don't Know | 51 | (15.2) | 25 | (14.9) | 26 | (15.5) |  |
| Diabetics should take extra care when cutting their toenails. | | **Yes** | 271 | (80.7) | 135 | (80.4) | 136 | (81.0) | .975 |
|  |  | No | 21 | (6.3) | 11 | (6.5) | 10 | (6.0) |  |
|  |  | Don't Know | 44 | (13.1) | 22 | (13.1) | 22 | (13.1) |  |
| Cuts and abrasions on diabetics heal more slowly. | | **Yes** | 289 | (86.0) | 145 | (86.3) | 144 | (85.7) | .977 |
|  |  | No | 24 | (7.1) | 12 | (7.1) | 12 | (7.1) |  |
|  |  | Don't Know | 23 | (6.8) | 11 | (6.5) | 12 | (7.1) |  |

*Note; This table presents item-level responses to the diabetes knowledge questionnaire, which* *was adapted from previously published and widely used diabetes knowledge instruments and KAP-based questionnaires reported in the literature. These items were used to construct the composite diabetes knowledge score analyzed in the main manuscript. Items were grouped into conceptual domains based on diabetes education frameworks to enhance interpretability; domain classification was applied for descriptive purposes only.* *Correct responses are indicated in bold. * p. value is based on Chi Squared test, #= p value based on Fisher Exact test as the assumptions of Chi Squared test were not met.*
